# Paraoxonase-1 as a Cardiovascular Biomarker in Caribbean Hispanic Patients Treated with Clopidogrel: Abundance and Functionality

**DOI:** 10.3390/ijms251910657

**Published:** 2024-10-03

**Authors:** Mariangeli Monero-Paredes, Ednalise Santiago, Kelvin Carrasquillo-Carrion, Jessicca Y. Renta, Igor B. Rogozin, Abiel Roche-Lima, Jorge Duconge

**Affiliations:** 1 Department of Pharmacology and Toxicology, School of Medicine, University of Puerto Rico, Medical Sciences Campus, San Juan, PR 00936, USA; mariangeli.monero@upr.edu; 2 Research Centers in Minority Institutions (RCMI) Program, Center for Collaborative Research in Health Disparities (CCRHD), University of Puerto Rico, Medical Sciences Campus, San Juan, PR 00936, USA; ednalise.santiago@upr.edu (E.S.); kelvin.carrasquillo@upr.edu (K.C.-C.); jessicca.renta@upr.edu (J.Y.R.); abiel.roche@upr.edu (A.R.-L.); 3 Computational Biology Branch, National Center for Biotechnology Information (NCBI), National Library of Medicine (NLM), National Institutes of Health (NIH), Rockville Pike MSC 3830, Bethesda, MD 20894, USA; ibrogozin@gmail.com; 4 Department of Pharmaceutical Sciences, School of Pharmacy, University of Puerto Rico, Medical Sciences Campus, San Juan, PR 00936, USA

**Keywords:** PON1, clopidogrel, Caribbean Hispanics, cardiovascular diseases, resistance

## Abstract

Clopidogrel, a prescription drug to reduce ischemic events in cardiovascular patients, has been extensively studied in mostly European individuals but not among Caribbean Hispanics. This study evaluated the low abundance and reduced activity of paraoxonase-1 (PON1) in clopidogrel-resistant patients as a predictive risk biomarker of poor responders and disease severity in this population. Thirty-six patients on clopidogrel (cases divided into poor and normal responders) were enrolled, along with 11 cardiovascular patients with no clopidogrel indications (positive control) and 13 healthy volunteers (negative control). Residual on-treatment platelet reactivity unit (PRU), PON1 abundance by Western blotting, and PON1 activity by enzymatic assays were measured. *PON1* genotyping and computational haplotype phasing were performed on 512 DNA specimens for two genetic loci (rs662 and rs854560). No statistical differences in mean relative PON1 abundance were found among the groups (*p* > 0.05). However, a significantly lower enzymatic activity was found in poor responders (10.57 ± 6.79 µU/mL) when compared to controls (22.66 ± 8.30 µU/mL and 22.21 ± 9.66 µU/mL; *p* = 0.004). PON1 activity among carriers of the most prevalent *PON1* haplotype (AA|AA) was significantly lower than in wild types (7.90 µU/mL vs. 22.03 µU/mL; *p* = 0.005). Our findings suggested that PON1 is a potential biomarker of cardiovascular disease severity in Caribbean Hispanics.

## 1. Introduction

Caribbean Hispanics are underrepresented in pharmacogenomics, which worsens health disparities in this diverse group. Clopidogrel, a common antiplatelet medication, helps prevent heart attacks, strokes, and peripheral artery disease, often combined with aspirin as a dual antiplatelet therapy (DAPT) [1]. While effective, clopidogrel shows a significant response variability due to factors like demographics, genetics, drug interactions, compliance, and comorbidities [2,3,4]. Clopidogrel response variability has become increasingly important, as it can be partially associated with poor clinical outcomes [5,6]. Around 33% of patients exhibit high on-treatment platelet reactivity (HTPR), increasing their risk for major adverse cardiac and cerebrovascular events (MACCEs) [7].

PON1 is a calcium-dependent enzyme associated with high-density lipoprotein (HDL) that protects against cardiovascular disease (CVD) by preventing low-density lipoprotein (LDL) oxidation and detoxifying organophosphates [8,9]. Consequently, PON1 has been described as a risk factor for developing coronary artery disease (CAD) [10]. The *PON1* p.Q192R polymorphism (rs662) is linked to enhanced enzyme function, while the L55M polymorphism (rs854560) is associated with reduced activity [9,11]. However, findings on p.Q192R’s impact are inconsistent, with some studies suggesting decreased enzymatic activity [12]. Some studies link PON1 activity to individual genotypes, while others postulate independence of genotypes [12,13]. This discrepancy may stem from population heterogeneity. Variability in allele frequencies, effect sizes, and linkage disequilibrium (LD) across populations may explain these discrepancies [14,15]. PON1 may also influence clopidogrel’s effectiveness [16].

This study aims to assess PON1 abundance and activity in Caribbean Hispanic patients who are poor responders to clopidogrel, using Western blot and enzyme assays. We tested the hypothesis that individuals with the *PON1* p.Q192R polymorphism, but not the p.L55M variant, will have higher plasma levels and enzymatic activity and a better clopidogrel response. Our approach involves assessing PON1 activity and genotypes in clopidogrel-treated patients and comparing them with controls.

## 2. Results

### 2.1. *PON1* Enzymatic Activity

**Figure 1:**
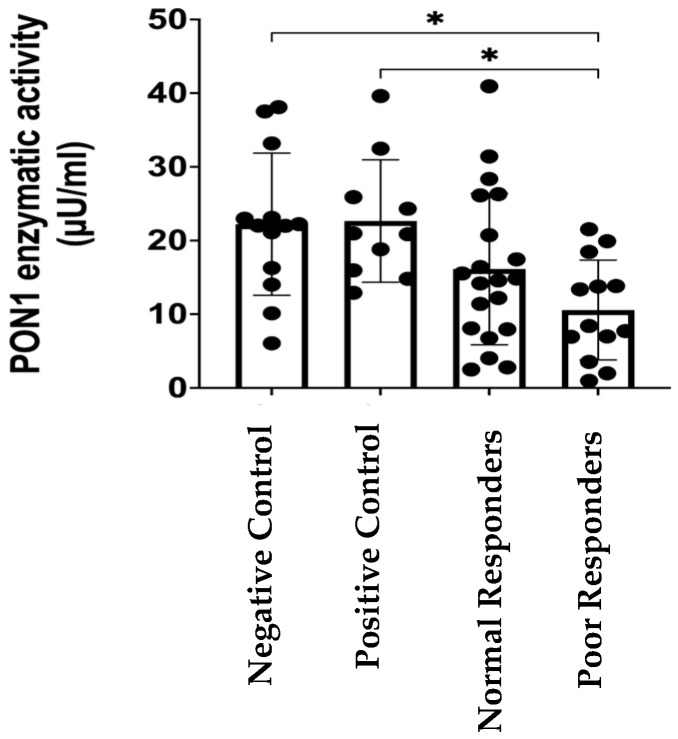
Enzymatic activity of PON1 (µU/mL) in plasma from participants in the control groups with and without cardiovascular diseases (i.e., positive and negative controls, respectively), normal and poor responders to clopidogrel. Note: Shapiro–Wilk test was run to determine normality of data distribution. One-way analysis of variance (ANOVA) was used to test significant differences among groups means (*p* = 0.0044). Tukey’s post hoc test was performed as multiple comparison (i.e., negative control versus poor responders, * *p* = 0.0101; positive control versus poor responders, * *p* = 0.0134). Dots represent enzyme activity per sample and bars are means of enzyme activity per group, with vertical bars representing ±SD.

Table 1 summarizes the demographic, genetic, and clinical data of participants in this study, including CYP2C19*2 minor allele frequency (MAF), comorbidities, clopidogrel indications (i.e., CAD; acute coronary syndrome, ACS; peripheral artery disease, PAD), rates of myocardial infarction (i.e., ST-Elevation Myocardial Infarction, STEMI, and Non-ST-Elevation Myocardial Infarction, NSTEMI, combined) occurrence, coronary artery stents, and co-medications for individuals on clopidogrel whose plasma samples were used to perform the PON1 enzyme activity assays (i.e., functionality). The groups were balanced, with data showing no significant difference between normal and poor responders, including their CYP2C19*2 MAFs, a well-known genetic marker of poor clopidogrel response (*p* = 0.35). Notably, the PON1 enzymatic activity among poor responders was significantly lower (10.57 µu/mL) than that in the negative control group (22.21 µU/mL, * *p* = 0.0134; Figure 1). Also, the poor responders’ group had a significantly lower enzymatic PON1 activity than that among the participants in the positive control group (22.66 µU/mL, * *p* = 0.0101; Figure 1). Despite being younger than the cases, the other baseline characteristics of the controls were comparable to those of the cases (i.e., 53.8% females with a body mass index, BMI, of 28.9 ± 6.2 among the negative controls without CVD; 54.5% females with a BMI of 30.1 ± 5.5 among positive CVD controls). 

### 2.2. *PON1* Relative Protein Abundance

**Figure 2:**
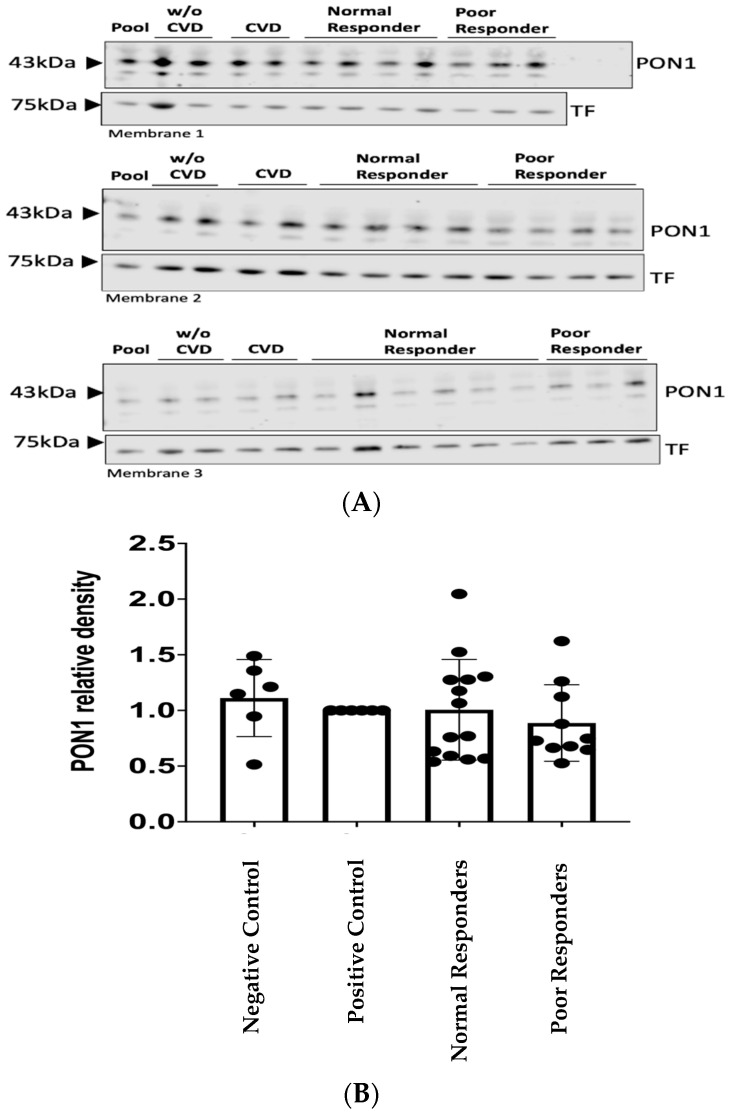
Western blot analysis for the validation of low abundance of PON1 protein in patients resistant to clopidogrel. (**A**) Representative membranes from a technical replicate of a Western blot analysis for PON1 protein in plasma samples from normal (n = 20) and poor responders (n = 16) to clopidogrel and the controls with (n = 11) and without cardiovascular diseases (n = 13). PON1 protein has a molecular weight of 43 kDa, while TF is 75 kDa. A pool control of all samples was included in each membrane to normalize the signal. (**B**) Densitometry analysis of PON1 protein relative abundance normalized to pool from three independent Western blots. Note: The comparison was made between the following groups: negative control (n = 13); positive control (n = 11); normal (n = 20); and poor responders (n = 16). Shapiro–Wilk test was performed to assess data distribution normality. One-way ANOVA was performed to test significant difference among the means of the groups (*p* = 0.6926). Values are the means with vertical bars representing ±SD.

**Figure 3:**
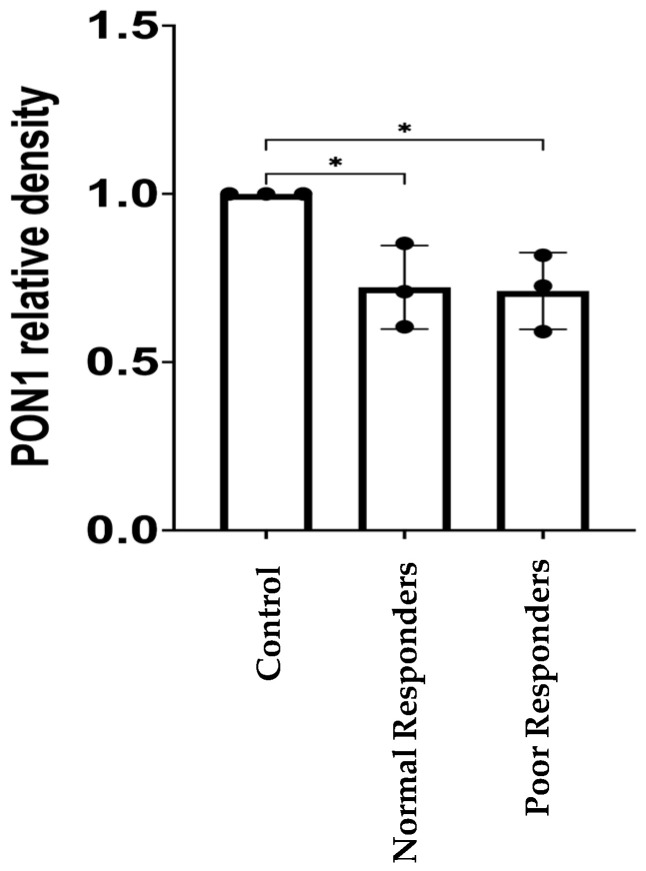
Densitometry analysis of PON1 protein relative abundance with the samples previously used in the proteomic study. Note: Each sample with a PON1 signal was normalized with the pool control signal from three independent Western blots. The samples used for this analysis (n = 9; representing the positive control and normal and poor responders, three samples each) were the same as from the previous proteomic studies where the PON1 protein was found to be downregulated among poor responders. Shapiro–Wilk test was performed to assess data distribution normality. One-way ANOVA was performed to test significant differences among the means of the groups (*p* = 0.0180). Tukey’s post hoc test was performed as a multiple comparison test (CVD versus normal responders, * *p* = 0.03; CVD versus poor responders; * *p* = 0.0256). Values are the means with vertical bars representing ±SD.

PON1 protein was identified with a band detected at 43 kDa (Figure 2A). The band of the loading control transferrin (TF) was detected at 75 kDa (Figure 2A). Our findings indicate no statistical differences between the groups means (i.e., negative control= 1.11; positive control= 1.00; normal responders= 1.01; poor responders= 0.89) when performing a validation of PON1 protein abundance for a combination of the initial sample set in the tandem mass tag–mass spectrometry (TMT-MS) and an independent sample cohort (Figure 2). However, when using the initial TMT-MS sample set (n = 9), there was a significant decrease (* *p* = 0.0180) in the relative abundance of PON1 protein among poor responders compared to the CVD control group (Figure 3, CVD = 1.00 and poor responders = 0.71; * *p* = 0.0256 post hoc test).

### 2.3. Genotypes and Haplotype Phasing

Table 2 summarizes the *PON1* genotypes and MAFs of the rs662 and rs854560 polymorphisms by groups (n = 60). For this study, we extracted the *PON1* genotypes specifically for the randomly selected 36 cases in our analysis, excluding the remaining reports in our database of 512 individuals (Database of Genotypes and Phenotypes, dbGaP Study Accession: phs003236.v1.p1). The *PON1* rs662 MAFs in poor and normal responders, as well as in the negative and positive control groups, were 53%, 57%, 42%, and 36%, respectively. On the other hand, *PON1* rs854560 MAFs for these groups were 19%, 32%, 15%, and 36%, respectively. No significant differences were found between groups for both variants (*p* = 0.4866 for the rs662 single-nucleotide polymorphism (SNP), and *p* = 0.4998 for the rs854560 SNP).

**Figure 4:**
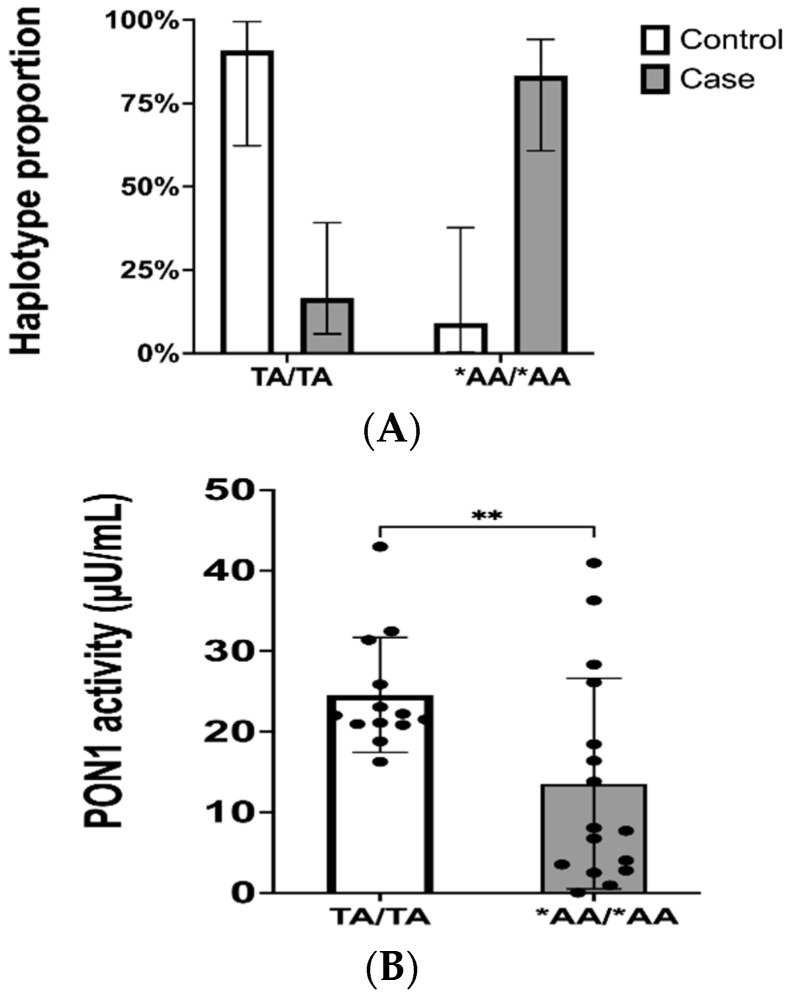
Association between TA|TA and *AA|*AA haplotypes and the severity of cardiovascular disease. (**A**) Haplotype proportions in controls (n = 24) and cases (n = 36). Haplotypes are presented as a proportion of the total number of participants, with vertical bars representing 95%CI. (**B**) PON1 enzymatic activity (µU/mL) of participants harboring the two most frequent haplotypes. ** indicates a highly statistically significant difference. Note: DNA base changes are represented with an asterisk (*). Dots represent enzyme activity per sample and bars are the means of enzyme activity per group, with vertical bars representing ±SD. For a statistical analysis to test the association between controls and cases, Fischer’s test was performed (*p* = 0.0001). A Shapiro–Wilk test determined the normality of PON1 activity data. A Mann–Whitney test was used to compare differences between groups (*p* = 0.0050). Individuals harboring the *AA/*AA haplotype are more likely to be poor responders (PRU > 230), as demonstrated in a previous work [17].

Genotype data are limited to only informing one whether a given patient is homozygous for the variant of interest (i.e., of carrier status), or heterozygous, without discriminating from which chromosome the variant came from. Determining haplotypes has a beneficial clinical implication as it can predict the severity of a disease. Therefore, applying haplotype phasing based on computational algorithms allows us to estimate which haplotype a patient will have by assigning alleles to chromosomes. We observed a higher frequency of the TA|TA haplotype (no variations) in the control groups, while the experimental groups had a higher frequency of the *AA|*AA haplotype (*—asterisk represents the base allelic variant) that carries the variant allele on the rs662 locus. A comparison of enzymatic activity by haplotypes showed a significant difference among the groups (* *p* = 0.0372). A comparison was made between controls and cases for the TA|TA vs. *AA|*AA haplotype. We found an association between haplotypes and the severity of cardiovascular disease (Figure 4A; ** *p* = 0.0001). In Figure 4B, a comparison of PON1 enzymatic activity between the two haplotypes showed a significantly (* *p* = 0.0050) lower enzymatic activity for those individuals harboring the haplotype with the rs662 variant in both chromosomes (*AA|*AA). Therefore, the *AA|*AA haplotype was found more frequently among patients with more severe cardiovascular diseases (i.e., ACS, stable CAD, or PAD patients).

## 3. Discussion

A TMT-MS proteomic analysis was performed on these Caribbean Hispanic patients on clopidogrel, to preliminarily identify circulating risk biomarkers that predict resistance to clopidogrel (response variability) and severity of CVD [18]. This study revealed that PON1 is downregulated in the plasma of patients who are resistant to clopidogrel (i.e., a low abundance among poor responders: −49.50) when compared to other cardiovascular controls without an indication for clopidogrel [18].

This is relevant because PON1 has previously been related to atherosclerosis and clopidogrel bioactivation pathways [8,16]. PON1 cysteine-284 interacts with oxidized LDL to reduce the accumulation of oxidized LDL in the sub-endothelial layer and prevent atherosclerosis progression. Therefore, PON1 has been described as a risk factor for developing cardiovascular diseases [9,19,20,21,22,23]. In this study, we validated PON1 protein in the initial sample set from the TMT-MS, where PON1 had a lower abundance in patients who were resistant to treatment with clopidogrel. These results suggest that PON1 could be a possible predictive biomarker of clopidogrel resistance in this population. A potential mechanism for this observation is that PON1 is participating in the biotransformation step of clopidogrel into the active metabolite. Therefore, due to a lower abundance of PON1 in patients from the poor responders’ group, they show a higher platelet reactivity, meaning that the clopidogrel active metabolite is not adequately inhibiting platelet aggregation. This is supported by a study where PON1 was identified as a rate-limiting step for the conversion of clopidogrel into its active metabolite, with the *PON1* p.*Q192R* polymorphism as a higher metabolite yielder [16]. Hence, this could suggest that the lower abundance of the PON1 protein in this group is causing resistance to clopidogrel. However, multiple studies since then have failed to replicate these findings by Bouman et al. Therefore, these recent data outweigh the hypothesis that PON1 could be a rate-limiting step for clopidogrel bioactivation and that it could serve as a biomarker of resistance to treatment [24,25,26,27,28].

We also found that genotypes concerning the *PON1* p.L55M variant were not affecting the corresponding protein abundance in plasma. This finding differs from previous studies where the *PON1* p.L55M polymorphism was found to be associated with reduced levels of gene expression, and hence, lower protein abundance in serum [29]. These results also suggest that the concentration of PON1 is independent from genotypes of the L55M variant in this locus. A possible explanation may be the LD. LD is the non-random association of alleles at different loci; therefore, two SNPs in LD have a high probability of being inherited together in a population. It has been shown in the literature that an SNP associated with a particular feature is not necessarily the causative variant, [30] but rather, it is a close SNP that is in LD with the associated SNP. In addition, the allelic frequency can change in admixed populations, generating different genetic variants with LD. For example, the tag-SNP *CYP2C9* rs202201137 was found to be in LD with SNPs associated with low doses of warfarin [31]. This tag-SNP has only been found in the Puerto Rican population, and the haplotype was named CYP2C9*61. In our study, a potential explanation is that *PON1* p.L55M is not the tag-SNP or the genetic variant that is in LD with the causal variant of a decreasing concentration of PON1. Genetic polymorphisms within the regulatory region of this gene have been early identified to alter the expression levels of PON1 protein [19,32,33]. The promoter region −108C/T polymorphism has previously been associated with a reduced PON1 concentration in plasma [32]. Therefore, a further analysis of variants in the regulatory regions of the *PON1* gene is needed to elucidate whether protein abundance is affected.

The published methods recommend validating quantitative proteomic results (TMT-MS) by using samples from an independent cohort that is different from the one initially used to conduct the proteomic studies [34]. Western blotting is a standard procedure to confirm our previous TMT-MS findings [35,36,37]. In our PON1 validation study, we added a negative control group of subjects without any diagnosed CVD and new samples across all the groups under study. Our results showed no statistical differences among groups under our experimental conditions, and thus, the previously observed low abundance of the PON1 protein in patients who are resistant to clopidogrel was not validated by using additional samples from an independent cohort. However, a nominal but non-significant trend towards lower PON1 abundance was observed in this group (Figure 2). The low abundance of PON1 in the poor responders could also be a direct consequence of the severity of cardiovascular diseases. Most (80%) of the patients in the poor responders group reported having dyslipidemia; however, only 3% were in therapy with statins. This could promote atherosclerosis in this group of patients and have a negative impact on their clinical outcomes. Another factor that is affecting the poor responders is their BMI. The BMI for the normal responders falls under the category of overweight, while the BMI from the poor responders group falls under obesity. It has been demonstrated that obesity enhances atherosclerotic disease and stimulates inflammation [38]. Taken together, this evidence suggests that the risk factors for CVD create an imbalance of oxidant and antioxidant molecules leading to a possible state of systemic oxidative stress in the poor responders [39,40,41]. In addition, it is known that the HDL-associated PON1 protein can lower its protective capacity against atherosclerosis when exposed to inflammation [42]. These findings are consistent with previous studies showing that PON1 protein concentration is reduced in CVD [43,44,45,46]. Therefore, PON1 could serve as a possible predictive biomarker of the severity of the CVD.

Since *PON1* polymorphisms could affect the proteolytic capability of the enzyme, we investigated if PON1 activity was also decreased in the poor responders group. We have previously found a significant association between the *PON1* genotype status and PRU values in this study cohort [17]. Therefore, we already know that those showing a poor response to clopidogrel (PRU > 230) are more likely to harbor the *AA/*AA haplotype. However, no distinction was made between poor and normal responders for the association analysis, because no difference was found between these two subgroups with respect to PON1 activity (Figure 1). Our data showed an enzymatic activity in patients who are resistant to clopidogrel (i.e., poor responders) that is significantly lower than that in the control groups (Figure 1). This may suggest that PON1 in poor responders has a reduced ability to act as an antioxidant enzyme, further increasing the severity of cardiovascular disease. In addition, poor responders had a higher incidence of NSTEMI than normal responders. Moreover, patients in the CVD-positive control group had less severe conditions, such as controlled hypertension or valve prolapse. Taken together, our results provide further evidence in favor of the role of PON1 as a potential predictive biomarker of cardiovascular disease severity in Caribbean Hispanics.

Genotyping data showed an overall MAF of ~50% for the rs662 variant (p.Q192R), while rs854560 (p.L55M) had a MAF of 25.8%. Allele frequencies vary substantially from one ancestral group to another, causing a wide variance in their frequency distribution across populations. It has been reported that prevalence of p.Q192R is 60% for South African ancestry, 65% for East Asians, 43% for South Asians, and 31% for European ancestry; and that of p.L55M is 4% for East Asians, 14% for Africans, 19% for South Asians, and 38% for Europeans [47,48]. The rs662 and rs854560 polymorphism of *PON1* have been described in the literature as changing enzyme catalytic capability and modulating enzyme expression, respectively [11,49]. To see how the combination of both major *PON1* polymorphisms could affect enzymatic activity, we performed haplotype phasing. We obtained 11 estimated haplotypes among the without CVD, with CVD, normal responders, and poor responders. This finding is consistent with another study in a cohort of Latino mothers and their newborns, where authors found 32 different haplotypes comprising multiple combinations of *PON1* polymorphisms in both regulatory (−909C>G, −162A>G, and −108C>T) and coding regions (p.Q192R and p.L55M) [50].

The most frequent haplotype was TA|TA (without SNPs) among the control groups, while *AA|*AA (*rs662 SNP) was the most frequent among patients in treatment with clopidogrel. As mentioned earlier, patients in the CVD control group mostly have less severe cardiovascular conditions, while the groups of patients in treatment with clopidogrel display more serious conditions, including ACS. Therefore, our results show an association between the haplotypes and the severity of the disease. Contrary to our hypothesis, we found that patients with severe CVD and with the *PON1* p.*Q192R* polymorphism had a lower enzymatic activity than those patients with less severe CVD and without the SNP. Results from earlier studies have indicated that having *PON1* p.*Q192R* caused a higher enzymatic activity and as a result, a better clinical outcome [51]. However, the results are inconsistent, as opposing evidence has been reported where *PON1* p.*Q192R* has been associated with a lower enzymatic activity and linked as a CVD risk factor [10,12]. The latter result reiterates what we have found in this study. The reported literature differences in PON1 activity can be a result of a causal variant in LD with *PON1*-rs662. In addition to supporting our results, Mackness et al. showed that a combination of PON1 protein concentration and activity is reduced in patients with Coronary Heart Disease (CHD) [13]. However, they did not find an association of the genotype with PON1 concentration and activity [13]. Our results differ, since we explored beyond the *PON1* p.Q192R genotype to determine the haplotype among groups and observed an association of the haplotype *PON1* p.Q192R and enzymatic activity. This is supported by studies that found an association of the *PON1* p.Q192R polymorphism with CHD [12].

Estrogens play a relevant role in PON1 abundance and activity. Postmenopausal women have reduced PON1 activity, and estrogen replacement therapy reverses these effects [52]. However, we do not anticipate a significant role of varying estrogen levels as a confounder in our study, because sex as a variable was not found to be significantly different between groups (*p*-value: 0.5152). Instead, women were fairly distributed in similar proportions between poor and normal responders. Furthermore, age did not vary significantly between groups (67 ± 10; *p*-value: 0.9817); therefore, no differences in the relative proportion of pre- and postmenopausal women between groups are expected. This study has some limitations, including the lack of a large external validation cohort, control by multiple covariates and potential confounders. Moreover, the relatively small sample size may limit the generalizability of the findings from a clinical perspective. Finally, while statins can influence PON1 activity, our analysis found no significant differences in statin use between poor and normal responders (*p* = 0.2036). However, we recognize that not stratifying by statin use might be considered a limitation.

Studies have demonstrated a relationship between carrying the *PON1* p.L55M variant and having more susceptibility to CAD [53,54]. On the contrary, our results showed that *PON1* p.L55M was neither associated with the disease nor its activity. This could be explained by the argument that different populations have a different expression of genes and epigenetic changes influenced by environmental risk factors such as diet and exercise [55,56,57,58]. This idea is supported by other studies, where populations yield inter-variability with respect to *PON1* polymorphism distribution and the alteration of its phenotype by consequence [48,59,60].

## 4. Materials and Methods

### 4.1. Study Population

Plasma specimens from thirty-six cardiovascular patients on clopidogrel (75 mg/day for at least 6 months), alone or as part of DAPT for ACS, stable CAD, or PAD, were used for the experimental procedures described in the study. These patients were randomly selected from a larger study cohort of 512 participants in a clinical protocol (Clinical Trial Registration Unique Identifier: NCT03419325). Furthermore, additional plasma samples from thirteen healthy volunteers (i.e., without CVDs) and eleven cardiovascular patients without an indication for clopidogrel were also used as negative and positive controls, respectively. All participants were originally recruited between January 2018 and July 2020 at two medical facilities in the Commonwealth of Puerto Rico (i.e., University Hospital at Carolina, Cardiovascular Center of Puerto Rico and the Caribbean). Patients who met the inclusion/exclusion criteria (Table 3) and signed an institutional review board (IRB)-approved written broad informed consent for future studies (protocol number A4070417) were enrolled. Demographical and clinical data were collected from medical records and/or during interviews, along with blood samples in two 3.0 mL 3.2% sodium citrate tubes. 

### 4.2. Sample Processing

The collected blood samples from the participants were used to measure PRU in the plasma using the VerifyNow^®^ ex-vivo P2Y12 platelet function assay (Accumetrics Inc., San Diego, CA, USA). The individual results of this test were used to identify poor (PRU ≥ 230) or normal (PRU < 230) responders to clopidogrel. For DNA isolation, 200 µL of blood was placed in a 2 mL Eppendorf tube, and samples were processed in the QIAcube following the QIAamp DNA Blood Mini Kit Protocol from QIAGEN (QIAGEN Inc., Valencia, CA, USA). DNA quantification was performed using the NanoDrop^TM^ 2000 Spectrophotometer (Thermo Fisher, Waltham, MA, USA) and samples were stored at −20 °C. To obtain plasma samples, blood was centrifuged at 3000 rpm for 10 min and stored at −80 °C.

### 4.3. *PON1* Function Assay

The Paraoxonase 1 Activity Assay Kit protocol (ab241044; Abcam Inc., Waltham, MA, USA) was used to determine the enzymatic activity of PON1 (µU/mL) on each sample. Briefly, standards were prepared by diluting the Fluorescence Standard with the Paraoxonase Assay Buffer to obtain concentrations within a range from 0 to 1000 pmol/well. The plasma samples were diluted (1:10) in Paraoxonase Assay Buffer in each technical (n = 3) and biological replicate (i.e., negative controls, n = 13; positive controls, n = 11; normal responders, n = 20; and poor responders, n = 16). Additionally, each sample was combined with an Inhibitor Mix for each technical replicate (n = 3) with the purpose of specific activity validation. Background Mix was added as a negative control. A Positive Control Mix and a Positive Control Mix with the Inhibitor Mix were also present. All reactions were conducted in 96-well plates, and controls had technical replicates (n = 3). 

The 96-well plate was incubated for 10 min to allow the interaction between PON1 protein and its inhibitor. For all samples and controls, 20 µL of 5× PON1 substrate solution was added. A SpectraMax M3 instrument (VWR, Radnor, PA, USA) was used to read fluorescence (Ex/Em = 368/460) on the microplate for 1 h after adding the fluorogenic substrate. Data were obtained using SoftMax Pro v6.2.1 software. Standard curves were used to determine the amount of metabolites in each sample. PON1 enzymatic activity was then calculated with the amount of metabolite, the time change for a linear phase, the dilution factor, and the volume of each sample on the wells according to protocol (ab241044) [61].

### 4.4. Western Blot Analysis

For the Western blot analysis, the existing plasma samples from normal (n = 20), poor responders (n = 16), healthy volunteers (negative control, n = 13), and cardiovascular patients without an indication for clopidogrel (positive control, n = 11) were used. To determine the plasma protein concentration, we used the DC protein assay kit (Bio-Rad, Contra Costa County, CA, USA) and bovine serum albumin (BSA) as standard protein. A total of 25 µg of protein was quantified from the plasma samples and loaded into a 10% sodium dodecyl sulfate (SDS) polyacrylamide gel for electrophoretic separation, and the proteins were transferred from the gel into a 0.45 µm polyvinylidene fluoride (PVDF) membrane. The PVDF membranes were blocked with Intercept^®^ blocking buffer for an hour, followed by incubation with PON1 (4G8D3) mouse monoclonal antibody (dilution 1:1000; Santa Cruz Biotechnology, Dallas, TX, USA) overnight at 4 °C in a shaker. The membranes were incubated with the secondary antibody, anti-mouse IRDye 680RD antibody (1;7500; LI-COR, Lincoln, NE, USA), in the shaker at room temperature. For the loading control, TF (101) rabbit monoclonal antibody (dilution 1:1000; Thermo Fischer, Waltham, MA, USA) and goat anti-rabbit IRDye 680RD (1:15,000; LI-COR, Lincoln, NE, USA) were used. Due to possible inter-assay variability, we used a pool control to normalize the samples. Briefly, 25 µg of protein from the 36 samples was mixed as a pool and quantified for plasma protein concentration as described above. From the pool, 25 µg was loaded into each of the 10% SDS polyacrylamide gels, and the Western blot protocol was performed as mentioned above. The membranes were scanned in the Odyssey^®^ CLx Imager (LI-COR, Lincoln, NE, USA) in AUTO (680 nm and 800 nm channels), and the immune fluorescent bands were quantified with Image Studio^TM^ Software v6.0. Data were expressed relative to the cardiovascular group.

### 4.5. Genotyping Microarray

Genotyping microarrays were run on 512 DNA specimens from our biorepository using the Infinium^®^ Multi-Ethnic AMR/AFR BeadChip (Illumina^®^, San Diego, CA, USA) following the manufacturer’s protocol. Briefly, 4 µL of genomic DNA was used for amplification during an incubation period of 20 to 24 h. DNA was fragmented, precipitated, and re-suspended for the hybridization of the DNA to the BeadChip overnight. The DNA BeadChip was washed with PB1, and the DNA was extended and stained for imaging through the iScan instrument (Illumina®, San Diego, CA, USA). GenomeStudio v2.0.5 Software was used to analyze the results from the iScan instrument, using GRCh37 as the reference genome build.

### 4.6. Haplotype Phasing for *PON1* Gene 

Individual *PON1* haplotypes of variants rs662 and rs854560 were determined using the available data from the genotyping microarray and the Segmented HAPlotype Estimation and Imputation Tools (SHAPEIT) v2.0 software. The results from the Multi-Ethnic BeadChip were obtained as pedigree information (.ped) and variant information (.map) files by using the PLINK Report Plug-in v2.1.4 in GenomeStudio v2.0.5 software. The files were converted to VCF files through the PLINK v1.07 software [62]. Appropriate data wrangling was applied by using manipulating and managing tools such as bcftools v1.14, VCFftools v0.1.13, and command-line shell. SHAPEIT v2.0 software [63] was executed according to the protocol for an estimation of computational haplotype phasing for chromosome 7. The phased dataset was filtered for *PON1* variants rs662 (position-94937446) and rs854560 (position-94946084). The phased and filtered datasets were compressed, indexed, and normalized due to inverted reference and alternate alleles. Further details can be found in (Supplementary Materials).

### 4.7. Statistical Analysis

The sample size was calculated for Western blot analysis by using a one-way balanced ANOVA test from the R package pwr. Due to multiple comparisons, a Bonferroni correction was made to the alpha value of 0.05 to reduce the false positive rate (type I error). With an effect size of 1.1 and a significance level of 0.0125, a minimum of five (n = 5) plasma samples for each of the four groups were required to achieve 80% statistical power. The sample size was calculated for enzymatic activity assays in the same manner. For this purpose, with an effect size of 1.4 and a significance level of 0.0125, a minimum of four plasma samples (n = 4) were required to achieve 80% statistical power.

Statistical analyses were carried out by GraphPad Prism v9.4.0. After testing the normality of the data by the Shapiro–Wilks test, a one-way ANOVA was run as a variance test for group comparisons in the Western blot and PON1 activity results. Tukey’s post hoc test was applied if there was a significant difference between the means among the groups, to determine which group pair was statistically significant. Continuous variables were presented as mean ± SD. A chi-square test was performed to assess the genotype frequency. The 95% confidence interval (95% CI) was calculated using the Wilson–Brown method.

## 5. Conclusions

In conclusion, inclusion of minority populations in pharmacogenomics and pharmacoproteomic studies is limited. The necessity for identifying valid biomarkers in an admixed population is increasingly important, especially because of the high prevalence of cardiovascular disease. We have demonstrated that there is a trend towards lower PON1 abundance in plasma, along with its diminished enzymatic activity, among patients who are carriers of the rs662 polymorphism and resistant to clopidogrel. The results presented herein indicate that PON1 could potentially serve as a useful predictive biomarker of the cardiovascular disease’s severity in Caribbean Hispanic patients suffering from ACS or stable CAD. Our findings also provide the foundations for future studies on valid clinical “omic” biomarkers for CVD and help reduce the knowledge gap in cardiovascular pharmacogenomics/proteomics of the Caribbean Hispanic population. A larger study is warranted to further support our major findings and concluding remarks.

While clinically relevant outcomes (e.g., MACCEs) are indeed valuable, they are beyond the scope of this study. Instead, we opted to use a PRU-based classifier as a surrogate of these events, which allows us to draw valid conclusions within the constraints of our study’s design and resources. However, we recently demonstrated the benefits of using individual genotypes to reduce MACCEs among post-PCI Caribbean Hispanic patients and inform antiplatelet therapy (i.e., clopidogrel vs. a more potent, alternative antiplatelet drug) [64]. Despite the availability of more potent antiplatelet drugs, their increased bleeding risk necessitates careful patient selection and highlights the importance of personalized approaches to antiplatelet therapy (e.g., by identifying risk biomarker of poor outcomes and disease severity).

## Figures and Tables

**Table 1:** Demographics, genetics, and clinical data of Caribbean Hispanic controls (i.e., negative and positive) and cardiovascular patients on treatment with clopidogrel (75 mg/daily) who were donors of plasma specimens for PON1 enzyme activity assays and Western blot analyses.

Characteristics	All Patients (n = 36)	Normal Responders (n = 20)	Poor Responders (n = 16)	*p* Value	Negative Controls (n = 13)	Positive Controls (n = 11)
Age (mean ± SD)	67 ± 10	67 ± 9	68 ± 10	0.9817	48 ± 11	53 ± 18
Sex (%, n)				0.5152		
Female	56% (20)	50% (10)	63% (10)		53.8% (7)	54.5% (6)
Male	44% (16)	50% (10)	38% (6)		46.2% (6)	45.5% (5)
BMI	30.9 ± 5.9	29.6 ± 5.2	32.6 ± 6.5	0.1291	28.9 ± 6.2	30.1 ± 5.5
Current Smoker (%, n)	19% (7)	20% (4)	19% (3)	>0.9999	15.4% (2)	18.2% (2)
Diabetes Mellitus (%, n)	92% (33)	90% (18)	94% (15)	>0.9999		91% (10)
Dyslipidemia (%, n)	92% (33)	95% (19)	88% (14)	0.5742		91% (10)
Hypertension (%, n)	97% (35)	95% (19)	100% (16)	>0.9999		100% (11)
Clopidogrel Indication (%, n)						
Stable CAD or ACS	72.2% (26)	75% (15)	68.75% (11)			
PAD	27.78% (10)	25% (5)	31.25% (5)	0.7225		
MI, STEMI, and NSTEMI (%, n)	2.77% (1)	0% (0)	6.25% (1)	0.4444		
Coronary Artery Stents (%, n) ^#^	36.1% (13)	40% (8)	31.3% (5)	0.7314		
Aspirin Users (%, n)	64% (23)	60% (12)	69% (11)	0.7314		0% (0)
CCB Users (%, n)	31% (11)	35% (7)	25% (4)	0.7182		27.3% (3)
Cilostazol Users (%, n)	14% (5)	15% (3)	13% (2)	>0.999		
PPI Users (%, n)	28% (10)	20% (4)	38% (6)	0.2853		18.2% (2)
Statins Users (%, n)	81% (29)	90% (18)	69% (11)	0.2036		82% (9)
CYP2C19*2 Status (MAF, %, n) *	13.9% (10)	12.5% (5)	15.6% (5)	0.7426	15.4% (4)	13.6% (3)

Note: Body mass index (BMI), myocardial infarction (MI), calcium channel blockers (CCB), and proton pump inhibitors (PPI). Standard deviation (SD). Age in years old. BMI data are shown as kg/m^2^. Statistical analysis was performed to compare normal versus poor responders using Fisher’s exact test, Student’s *t*-test, and *z*-test for proportions. ^#^ All patients with ACS and some with stable CAD underwent PCI (i.e., percutaneous coronary interventions with stent deployment). * n refers to the total # of minor alleles detected in the study groups.

**Table 2:** *PON1* genotypes and MAFs at the two polymorphic sites of interest (rs662 and rs854560) in samples used to run the Western blot analysis.

Variable	Negative Controls (n = 13)	Positive Controls (n = 11)	Normal Responders (n = 20)	Poor Responders (n = 16)
rs662 (p.Q192R)				
QQ	4 (31%)	5 (45.5%)	3 (15%)	2 (12.5%)
QR	7 (54%)	4 (36.4%)	11 (55%)	11 (68.75%)
RR	2 (15%)	2 (18.1%)	6 (30%)	3 (18.75%)
MAF	42.31%	36.4%	57.5%	53.12%
95%CI	19.33–68.05	13.81–60.94	39.07–73.50	34.21–74.18
rs854560 (p.L55M)				
LL	9 (69.2%)	6 (54.5%)	10 (50%)	10 (62.5%)
LM	4 (30.8%)	2 (18.2%)	7 (35%)	6 (37.5%)
MM	0 (0%)	3 (27.3%)	3 (15%)	0 (0%)
MAF	15.38%	36.4%	32.5%	18.75%
95%CI	2.96–44.80	19.33–68.05	17.93–50.66	8.07–41.60

Note: Control groups are defined as negative (i.e., healthy volunteers) and positive (i.e., cardiovascular patients without an indication for clopidogrel) controls. *PON1* wild types are denoted by the amino acid glutamine (Q) in position 192 and leucine (L) in position 55. The rs662 and rs854560 polymorphisms are defined as a change of Q in position 192 to arginine (R) and L in position 55 to methionine (M), respectively. Genotype data are presented as counts and proportions (n, %). Chi-square tests were performed for the comparisons of the *PON1* rss662 and rs854560 MAFs between the groups. The 95% confidence intervals (95% CI) of the corresponding MAFs for these two SNPs in each group were calculated using the Wilson–Brown method.

**Table 3:** Inclusion and exclusion criteria for patients.

Inclusion Criteria	Exclusion Criteria
Caribbean Hispanics residing in Puerto Rico. Both sexes (i.e., males/females). Age ≥ 21 years-old. Receiving clopidogrel (75 mg/day) for therapeutic indications (ACS, stable CAD, PAD) over at least 6 months. No clinically active hepatic abnormality. The ability to understand the requirements of the study. The ability to comply with the study procedures and protocols. A female patient is eligible to enter the study if she is of child-bearing potential and not pregnant or nursing, or not of child-bearing potential.	Non-Hispanic patients Currently enrolled in another active research protocols Blood Urea Nitrogen (BUN) > 30 and creatinine > 2.0 mg/dL Hematocrit (Hct) ≤ 25% Nasogastric or enteral feedings Acute illness (e.g., sepsis, infection, anemia) HIV/AIDS, Hepatitis B patients Alcoholism and drug abuse Patients with any cognitive and mental health impairment Sickle cell patients Active malignancy Patients taking another antiplatelet

## Data Availability

For imputations and computational haplotype phasing, the data used as the reference populations are Surui and Karitiana in Brazil, Piapoco and Colombian in Colombia, and Maya and Pima in Mexico, found at https://www.internationalgenome.org/data-portal/data-collection/hgdp (accessed on 7 October 2021). IBS and YRI populations are from the NHGRI Sample Repository for Human Genetic Research of the Coriell Institute found at https://www.internationalgenome.org/data-portal/population (accessed on 7 October 2021). The datasets from the Caribbean Hispanics analyzed during the current study are available from the corresponding author upon request.

## Supplementary Materials

The following supporting information can be downloaded at: https://www.mdpi.com/article/10.3390/ijms251910657/s1.
