# Supplementary material for: Paraoxonase-1 as a Cardiovascular Biomarker in Caribbean Hispanic Patients Treated with Clopidogrel: Abundance and Functionality
Source: Int J Mol Sci. 2024 Oct 3;25(19):10657. doi: 10.3390/ijms251910657 (PMC11477108; doi:10.3390/ijms251910657)
Supplement: Supplementary file 1 [file ijms-25-10657-s001.zip › ijms-3203279-supplementary.pdf]

Supplementary Material (Appendix A)

Preparation and Execution of Haplotype Phasing Data

A. Data preparation

1. Each 8 sample Multi-Ethnic Beadchip AMR/AFR genotype data was obtained from Genome Studio Software, PLINK Report Plug-in v2.1.4 as a pedigree information (.ped) and variant information (.map) file. Samples included groups without CVD, CVD, normal PRU, and high PRU. The data was converted to a variant call format (.vcf) file for subsequent steps.

   ```
   ./Plink --ped DrDucongeClopidogrelBatch19.ped --map DrDucongeClopidogrelBatch19.map --recode vcf --out DrDucongeClopidogrelBatch19.vcf
   ```

   Output: The --recode vcf parameter transformed the genotype data into a VCF file. The VCF file contained columns such as chromosome (CHROM), position or base-pair coordinate (POS), ID, reference base (REF), alternate base (ALT), quality score of calls (QUAL), passing filter (FILTER), additional information (INFO), format of the genotype calls (FORMAT), and subsequent sample IDs.

2. Chromosomes 0 and 23 to 26 were removed from the dataset.

   ```
   ./Plink --vcf DrDucongeClopidogrelBatch19.vcf --not-chr 0 23-26 --double-id --recode vcf --out DrDucongeClopidogrelBatch19_Chr.vcf
   ```

   Output: The --not-chr parameter removed the chromosomes that were not autosomes.

3. vcf files were compressed for indexing purposes.

   ```
   bgzip DrDucongeClopidogrelBatch19_Chr.vcf
   ```

Ouput: Compressed vcf files in the format file.vcf.gz.

4. The compressed files were indexed using the tabix tool.

./bcftools tabix -p vcf DrDucongeClopidogrelBatch19_Chr.vcf.gz

Output: An index position file was created. This allows searching through big datasets.

5. VCF files were normalized, meaning that we used the referenced genome GRCh37 to left-align and joining biallelic sites into a multiallelic row (-m+) in the dataset. The normalization step allowed the correct positioning of the reference and alternate sequence allele for further analysis.

./bcftools norm -m+ -c ws -Oz -o DrDucongeClopidogrelBatch19_Chr_Norm.vcf.gz -f human_g1k_v37.fasta DrDucongeClopidogrelBatch19_Chr.vcf.gz

Output: The parameter -m+ caused joining of multiallelic sites in the datasets from batch 19. There were a total of 1,380,255 of reference and alternate alleles. From that quantity, a 141,411 were modified and 639,104 were added.

6. The normalized compressed VCF files were indexed using the tabix tool to create a new indexed file of genome positioning:

./bcftools tabix -p vcf DrDucongeClopidogrelBatch19_Chr_Norm.vcf.gz

Output: An index position file was created. This allowed searching through big datasets.

7. Steps one (1) to six (6) were repeated accordingly to the quantity of batches analyzed. In this case it was repeated for batch 20 and 21. Once all individual batches were normalized and indexed, we proceeded to the merging of the VCF files.

8. Batch 19, 20, and 21 were merged to a single VCF by ID (-m id):
/bcftools merge -m id DrDucongeClopidogrelBatch19_Chr_Norm.vcf.gz DrDucongeClopidogrelBatch20_Chr_Norm.vcf.gz DrDucongeClopidogrelBatch21_Multi_Chr_Norm.vcf.gz -Ov -o ClopidogrelBatches19to21Multi_Merged_OFFICIAL_Norm.vcf
Output: A new VCF file was created containing all samples from batch 19 to 21.

9. ID labels were modified from sentrix position to PPR:
./bcftools reheader -s sample_ids_merged19to21.txt -o ClopidogrelBatches19to21Multi_Merged_OFFICIAL_Norm _Header.vcf ClopidogrelBatches19to21Multi_Merged_OFFICIAL_Norm.vcf
Output: A VCF file with the PPR ID per sample.

10. Identified and kept the 26 samples or individuals of interest from the VCF file:
./vcftools --vcf ClopidogrelBatches19to21Multi_Merged_OFFICIAL_Norm_ Header.vcf --keep Samplestokeep.txt --recode --out ClopidogrelBatches19to21Multi_Merged_OFFICIAL_Norm _Header_26samples
Output: The parameter --kept filtered the 26 out of 69 PPR individuals listed in a plain text (.txt) file and maintained them in the VCF file.

11. Merging VCF file of batch 19 to 21 and VCF file from batch 1 to 17

a. VCF files were compressed and indexed as mentioned in previous step A.3 and A.4:

```
bgzip ClopidogrelBatches19to21Multi_Merged_OFFICIAL_Norm
_Header_26samples.vcf

bgzip ClopidogrelBatches1to17_Merged_OFFICIAL_Norm.vcf
```

Output: Two (2) compressed separate vcf.gz files were created.

```
./bcftools tabix -p vcf  ClopidogrelBatches19to21Multi_Merged_OFFICIAL_Norm
_Header_26samples.vcf.gz

./bcftools tabix -p vcf ClopidogrelBatches1to17_Merged_OFFICIAL_Norm.vcf.gz
```

Output: Two (2) index position files were created.

b. Both indexed and compressed VCF files were merged as previous step A.8:

```
./bcftools merge -m id ClopidogrelBatches1to17_Merged_OFFICIAL_Norm.vcf.gz
ClopidogrelBatches19to21Multi_Merged_OFFICIAL_Norm _Header_26samples.vcf.gz
-Ov -o ClopidogrelBatches1to21Multi.vcf
```

Output: A new uncompressed VCF file was created containing all samples from batch 1 to 17 and 19 to 21. A total of 537 individuals were kept.

12. Prior to haplotype phasing, the dataset needed to be split by chromosome. Since we focused on the haplotype for *PON1* gene located in chromosome 7, we used the parameter --chr 7 to filter by chromosome 7:

./vcftools --vcf ClopidogrelBatches1to21Multi.vcf --chr 7 --recode --out ClopidogrelBatches1to21Multi_Chr7.vcf

Output: A new VCF file was created with genotypes only for chromosome 7. A total of 74,573 of 1,380,255 sites were kept.

13. Identified and kept the 60 samples or individuals of interest from the VCF file as done previously in step A.10:

./vcftools --vcf ClopidogrelBatches1to21Multi_Chr7.vcf --keep 60Samples.txt --recode --out ClopidogrelBatches1to21Multi_Chr7_60Samples

Output: A new VCF file was created containing the 60 out of 537 individuals. The individuals of interest were listed in the text file.

14. The VCF file was converted into a a PLINK BED/BIM/FAM format for further analysis:

./plink --vcf ClopidogrelBatches1to21Multi_Chr7_60Samples --recode --make-bed --double-id --out ClopidogrelBatches1to21Multi_Chr7_60Samples

Output: BIM file was loaded with74573 variants, FAM file was loaded with 60 individuals, and BED file with the genotype calls.

B. Haplotype phasing using SHAPEIT v2 software

1. Haplotype phasing analysis was performed. The parameters --force --input-bed were used to indicate the unphased genotype data in the format of BED/BIM/FAM. The parameter --input-map was used to provide with a map of SNPs positions in centimorgan (cM) and offered recombination rates. The following command line estimated the haplotype for chromosome 7 variants:

```
./shapeit --force --input-bed ClopidogrelBatches1to21Multi_Chr7_60Samples.bed
ClopidogrelBatches1to21Multi_Chr7_60Samples.bim
ClopidogrelBatches1to21Multi_Chr7_60Samples.fam --input-map
genetic_map_chr7_combined_b37.txt --output-max
ClopidogrelBatches1to21Multi_Chr7_60Samples.phased.haps
ClopidogrelBatches1to21Multi_Chr7_60Samples.phased.sample
```

Output: The haplotype estimation was created as an output file using the parameter --output-max.

2. The phased file was used for the parameter --input-haps and converted to a VCF file using the parameter -convert and --output-vcf:

```
./shapeit -convert --input-haps ClopidogrelBatches1to21Multi_Chr7_60Samples.phased --
output-vcf ClopidogrelBatches1to21Multi_Chr7_60Samples.phased.vcf
```

Output: A VCF file was created with the phased genotypes for chromosome 7.

C. PON1 variants rs662 and rs854560 haplotype

1. The phased data was obtained for the *PON1* positions 94937446 (rs662) and 94946084 (rs854560) by using the grep command, where F searched for fixed strings, w searched for the specific whole word, and f specified the file where to obtain the patterns.

   Grep -Fwf Positions_rs662_rs854560.txt
   ClopidogrelBatches1to21Multi_Chr7_60Samples.phased.vcf >
   ClopidogrelBatches1to21Multi_Chr7_60Samples_PON1Variants.phased.vcf

   Output: A new VCF file was created with the phased data for PON1 rs662 and rs854560 variants.

2. Since the newly created VCF file from previous step B.3 does not have headers, a new header was placed using the following commands.

   a. Original header before filtering for the *PON1* variants was obtained:

      head -n6 ClopidogrelBatches1to21Multi_Chr7_60Samples.phased.vcf >
      Header_ClopidogrelBatches1to21Multi_Chr7_60Samples.phased.vcf

      Output: A VCF file was created containing the header for the first six (6) columns.

   b. The VCF file containing the header for the first six (6) columns was concatenated with the VCF file containing the phased PON1 variants using the cat command:

      cat Header_ClopidogrelBatches1to21Multi_Chr7_60Samples.phased.vcf
      ClopidogrelBatches1to21Multi_Chr7_60Samples_PON1Variants.phased.vcf >
      ClopidogrelBatches1to21Multi_Chr7_60Samples_PON1Variants_Header.phased.vcf

Output: The final VCF file with the header and phased PON1 variants rs662 and rs854560 was created.

3. Normalization of the phased data was performed due to inverted reference and alternate allele.
    a. File was compressed for indexing purposes, as previous step A.3:
    ```
    Bgzip
    ClopidogrelBatches1to21Multi_Chr7_60Samples_PON1Variants_Header.phased.vcf
    ```
    Ouput: Compressed VCF files in the format vcf.gz.

    b. File was indexed, as previous step A.4:
    ```
    ./bcftools tabix -p vcf
    ClopidogrelBatches1to21Multi_Chr7_60Samples_PON1Variants_Header.phased.vcf.
    gz
    ```
    Ouput: An index position file was created.

    c. Compressed and indexed VCF file was normalized, as previous step A.6:
    ```
    ./bcftools norm -m+ -c ws -Oz -o
    ClopidogrelBatches1to21Multi_Chr7_60Samples_PON1Variants_Header_Norm.vcf.
    gz -f human_g1k_v37.fasta
    ClopidogrelBatches1to21Multi_Chr7_60Samples_PON1Variants_Header.phased.vcf.
    gz
    ```

Output: The final VCF file with the header and phased *PON1* variants rs662 and rs854560 was corrected for inverting the reference and alternate allele.
